# The Effect of Cooking and Cooling Chickpea Pasta on Resistant Starch Content, Glycemic Response, and Glycemic Index in Healthy Adults

**DOI:** 10.3390/metabo14110585

**Published:** 2024-10-28

**Authors:** Adrianna Bojarczuk, Paulina Kęszycka, Krystian Marszałek, Danuta Gajewska

**Affiliations:** 1 Department of Fruit and Vegetable Product Technology, Prof. Wacław Dabrowski Institute of Agricultural and Food Biotechnology, State Research Institute, 36 Rakowiecka Street, 02-532 Warsaw, Poland; krystian.marszalek@ibprs.pl; 2 Department of Dietetics, Institute of Human Nutrition Sciences, Warsaw University of Life Sciences (WULS), 159C Nowoursynowska Street, 02-776 Warsaw, Poland; paulina_keszycka@sggw.edu.pl

**Keywords:** chickpea pasta, resistant starch, cooking and cooling, postprandial glycemia, glycemic index

## Abstract

**Background/Objectives:** Legume seeds, such as chickpeas, are a rich source of resistant starch (RS) and have a low glycemic index (GI). The aim of this study was to evaluate the effect of cooking and cooling chickpea pasta on the RS content, glycemic response, and GI in healthy subjects. **Methods:** Twelve healthy subjects of both sexes, aged 18–65 years, participated in this study. Each person consumed two standardized portions of chickpea pasta: (i) freshly cooked (FCP) and (ii) cooked chickpea pasta which was cooled for 24 h at 4 °C and reheated before consumption (CCP). Glucose solution was provided as a reference food. Participants consumed chickpea pasta in a random order. GI measurements were completed using the standard methodology and calculated according to the ISO 2010 standard. **Results:** The cooling and reheating process significantly increased the RS content of boiled chickpea pasta (from 1.83 g/100 g to 3.65 g/100 g) and had a beneficial effect on postprandial glycemia in healthy individuals. The CCP pasta had a significantly lower GI value than the FCP pasta (33 vs. 39, *p* = 0.0022). A significant difference in the glucose, as identified by the incremental area under the curve (IAUC), was observed between the CCP and FCP (1327.9 ± 414.8 mg/dL/min vs. 1556.1 ± 456.9 mg/dL/min, *p* = 0.0022). The cooling–reheating process did not affect the sensory attributes of the chickpea pasta. In general, the overall acceptability of the CCP pasta was similar to that of the FCP pasta. **Conclusions:** The results of our study support the hypothesis that a reduced glycemic response after simple changes in technological intervention leads to a decrease in postprandial blood glucose and GI. This can be helpful for people who need to control postprandial glycemia.

## 1. Introduction

A plant-based diet (PBD) is becoming increasingly popular worldwide [1]. A well-planned, nutritionally adequate PBD is recognized as healthy and sustainable; therefore, the EAT-Lancet Commission strongly advocates for the implementation of more plant-based diets globally. Many national food-based dietary guidelines promote a higher intake of plant foods and recommend substituting animal products or animal proteins with plant-based foods/plant-based proteins [2]. However, according to the study by Klapp et al. [3], only 40% of national food-based dietary guidelines contain a position on vegetarian diets, and 18% of them do not mention plant-based sources of protein.

PBDs are based on grains, vegetables, fruits, seeds, nuts, and legumes. The latter are the second most important group of plants in human nutrition, after cereals. Legumes are considered a rich source of proteins, carbohydrates (up to 65%), soluble and insoluble fibers, minerals, vitamins (niacin, thiamine, and riboflavin), and plant sterols. The beneficial effects of legume intake on carbohydrate and lipid metabolism, weight loss, and appetite reduction, as well as its capacity to reduce the risk of certain cancers and improve overall diet quality, have been demonstrated [2,4]. These effects are associated with the high content of macro- and micronutrients and phytochemicals and the lower energy density of legumes. In addition, legume seeds have a high satiety index, which can be helpful in weight reduction, are naturally gluten free, and have a low glycemic index (GI) and glycemic load (GL) [5,6,7].

The GI is a system of ranking foods containing carbohydrates based on the postprandial blood glucose response, compared with a reference food [8,9]. The GI provides useful information for diverse groups of people who have to control their blood glucose levels for the prevention or management of disease. Low-GI diets have been reported to protect against several metabolic diseases like obesity, metabolic syndrome, diabetes, and cardiovascular disease [10].

The most important influence on the GI value is the carbohydrate content of the food. Starch is the main source of carbohydrates in the human diet, and the process of its digestion and absorption affects the glycemic response. A part of starch is not digested and is absorbed in the small intestine before it passes into the large intestine, where it is fermented by the intestinal microbiota. This type of starch is called resistant starch (RS). Being resistant to enzymes, this starch is not absorbed, retaining its function as dietary fiber. Many factors can affect the RS content of products, including plant species, forms of cultivation, the type of starch, and the coexistence of other nutrients or product processing and storage forms [11,12]. The cooling process of cooked starchy products leads to a decrease in the availability of digestible carbohydrates by producing RS in a process called retrogradation. Consumption of products with increased RS content may have a beneficial effect on postprandial glycemia due to slower digestion compared to food with a higher content of rapidly digested starch [13,14]. This may be relevant in the prevention and treatment of carbohydrate metabolism disorders, such as insulin resistance and diabetes, as changing some of the digestible starch into RS may decrease the GI and have a positive effect on postprandial glycemia [15,16]. There is a growing demand for innovative food products with low GI that are both healthy and environmentally friendly. A great example of this trend is legume pasta such as chickpea pasta, which combines high nutritional value with a low environmental impact.

There is a lack of scientific reports on the effect of retrograded starch from chickpea pasta on postprandial glycemia and the GI in healthy individuals. The current study was designed to analyze the impact of different methods of preparing chickpea pasta on RS content. We hypothesized that the process of cooling and reheating chickpea pasta would increase the amount of RS and thereby reduce postprandial glycemia and the GI in healthy adults. Thus, the aim of this study was to assess the effect of cooking and cooling and reheating chickpea pasta on the RS content, glycemic response, and GI in healthy subjects.

## 2. Materials and Methods

### 2.1. The Study Group

Fourteen people applied for the study. Twelve adults, constituting healthy volunteers of both sexes with normal body weight (BMI 18.5–24.9 kg/m^2^), met the selection criteria and were recruited for the study. The sample size was determined based on the protocol for the determination of the GI [9]. The recruited participants were students, graduates, and employees of the Warsaw University of Life Sciences. The inclusion criteria were as follows: age over 18 years and under 65 years; no diagnosed metabolic diseases; not using medications or dietary supplements affecting carbohydrate metabolism; no diagnosed food allergies or intolerances; and normal body weight. The exclusion criteria were as follows: pregnancy or lactation; age below 18 and above 65 years; underweight (BMI < 18.5 kg/m^2^) or overweight (BMI > 25 kg/m^2^); having a diagnosed metabolic disease; currently taking any medications or dietary supplements affecting carbohydrate metabolism; and food allergies or food intolerances. The qualification for the study was provided by healthcare professionals; a medical interview was conducted by a physician (10 min) and a nutritional interview was conducted by a dietitian (15 min).

All procedures involving human subjects were conducted according to the guidelines laid down in the Declaration of Helsinki. The study protocol was approved by the Rector’s Ethics Committee of Research with Human Participation—SGGW (Resolution No. 5/RKE/2023/U).

### 2.2. Study Protocol

The study was single-blinded and randomized. Participants were asked to attend a screening meeting and six meetings during the study. There were two break days between each test day. During the screening visit, all participants were informed of the study’s protocol and procedures and provided written informed consent. For each person, body height (using a SECA 213 Growth Meter), body weight, and body composition (using a TANITA BC-545N body composition analyzer) were measured. The BMI of individuals was calculated using the standard formula [weight (kg)/height × height (m^2^)] [17]. All measurements were carried out at the SGGW Dietary Clinic by a trained dietitian.

Each participant consumed the reference food and two tested foods: (i) freshly boiled chickpea pasta (FCP); and (ii) chickpea pasta that was boiled, cooled, and reheated after being stored at 4 °C for 24 h (CCP). Each test day, a single sample (reference food or pasta) was tested by all participants. The glucose solution was chosen as the reference food (50 g of glucose in 250 mL of water). The reference food was tested twice in two different sessions. On the following days, participants consumed test meals, but they were not informed about what type of pasta would be served during each session. They consumed pasta in a randomized sequence during non-consecutive days after an overnight fast. Meal consumption began between 8 and 8:30 a.m., and all participants were instructed to consume it at a relatively constant eating time (no less than 12 min and no more than 15 min). Participants were also instructed to consume the same meal for dinner and to refrain from performing any strenuous physical activity on the day before each of the 6 days of the study.

Capillary blood samples were collected at 0 (fasted state) and 15, 30, 45, 60, 90, and 120 min after glucose/tested food ingestion.

### 2.3. Test Meals

A commercial chickpea pasta, fusilli-shaped (Makarony Bartolini-P.P.H. Tabit Sp. z o.o., Wiązowna, Poland), was purchased from the local supermarket. This company’s pasta was selected because it contains 100% chickpea flour without additives, which allows for an accurate assessment of the ingredient’s impact on the analyzed parameters without interference from processing-related changes. In addition, this chickpea pasta is popular among consumers and readily available on the market throughout the country.

All participants consumed the same amount of chickpea pasta, containing 50 g of available carbohydrates. The total content of available carbohydrates had been previously determined and calculated using the analytical method. The available carbohydrate content of 100 g of dry pasta was 53.5 g; therefore, the calculated serving size of pasta to provide 50 g of available carbohydrates was 93.5 g. Water (250 mL) was offered during GI testing.

Chickpea pasta was prepared via two methods:

1. Cooked in 100 g/100 mL of unsalted boiling water for 15 min, drained, and served directly to the subjects (FCP);
2. Cooked (in 100 g/100 mL of unsalted boiling water for 15 min), drained, cooled rapidly (using cold water), and stored at 4 °C for 24 h prior to the consumption by participants. To ensure blinding, before being served to the subjects, the cooked and cooled pasta was reheated in water (100 °C, 3 min) to the same temperature as the fresh pasta (CCP).

### 2.4. Analytical Methods

Raw chickpea pasta samples were analyzed for total carbohydrates, protein, fat, ash, fiber, and water content using standardized methods. Moisture content was determined via the weight method (drying 180 min at 105–107 °C) [18]. Total protein content was analyzed via the Kjeldahl method according to PN-A-04018:1975/Az3:2002 [19]. Total fat content was analyzed according to PN-A-79011-4:1998 [20]. Total dietary fiber was analyzed according to 991.43 AOAC, 32-07 AACC [21,22]. Total ash content was estimated via the weight method (after roasting) according to EN ISO 2171:2010 [23]. The content of total carbohydrates was calculated according to EP and Council Regulation (EU) No. 1169/2011 of 25.10.2011. Carbohydrates were calculated based on the content of dry matter, ash, protein, fat, and fiber, based on the following formula: Total carbohydrates = [100 − % (Protein + Fat + Moisture + Ash + Fiber)] [19,20,21,22,23].

Both FCP and CCP were analyzed for resistant starch content using the AOAC 2002.02 method [24]. All analyses, with the exception of the carbohydrate content, were performed twice.

### 2.5. Glucose Measurements

Prior to the measurements, all study participants were properly instructed on using the equipment and taking measurements, including self-puncture of the fingertip and the hygiene rules associated with the violation of skin coverings. All measurements were supervised by healthcare professionals. Each time, 0.3–0.4 µL of capillary blood was required for testing. Glucose determinations in capillary blood were performed via the dry enzymatic method using test strips and Contour^®^ plus glucometers (Ascensia Diabetes Care, Warsaw, Poland Sp. z o.o.). Capillary blood was collected after disinfecting the fingertip, using disposable lancets.

### 2.6. Glycemic Index Determination

To determine the glycemic index of tested products, measurements were made of capillary blood glucose levels after consuming a solution containing 50 g of glucose (anhydrous glucose powder dissolved in 250 mL of water) as a standard and after consuming the test products in an amount that provided 50 g of available carbohydrates. Measurements were made in 2 repetitions at fasting, every 15 min for the first hour, and then at 90 and 120 min after the test. GI values were calculated by comparing the value of the area under the glycemic curve of the test product to the value of the area under the glycemic curve of the standard and multiplying the result by 100, according to the ISO 2010 standard [1]. For each subject, the GI was calculated as follows:

$$\mathrm{GI}*=(\frac{iAUCtest\;food*}{iAUCglucose\;standard})\times 100.$$

* GI—glycemic index; iAUC—incremental area under the blood glucose response curve.

The ratio between the individual incremental area under the glucose curve (iAUC) after consuming the reference food and the pasta samples was calculated for each participant. The glycemic index (GI) for each pasta type was then determined by averaging these ratios across all participants. The iAUC was calculated using the conventional trapezoidal method [1].

### 2.7. Sensory Assessment

Sensory evaluation of the cooked chickpea pasta was carried out using the 7-point hedonic scale, where a score of 1 indicated that the product was strongly disliked, and a score of 7 indicated that it was highly liked. All subjects were asked to evaluate such parameters as visual appearance, taste, smell, texture, and overall acceptability.

### 2.8. Statistical Analysis

Data were checked for normality using the Shapiro–Wilk test. Continuous variables are described as mean ± standard deviation (SD). Blood glucose response was analyzed using a two-way repeated-measures ANOVA, and area under the curve was analyzed using a one-way repeated-measures ANOVA. Pairwise comparisons were conducted using a Bonferroni post hoc correction. The differences between sensory properties were analyzed by using the related-samples Wilcoxon signed rank test. Statistical analyses were conducted using STATISTICA 13.1 (TIBCO Software Inc., Palo Alto, CA, USA). The significance level was *p* < 0.05.

## 3. Results

### 3.1. Study Group

The study group had a body mass index (BMI) within the normal range (22.5 ± 1.9 kg/m^2^) and optimal fasting blood glucose levels of 87.33 ± 4.7 mg/dL, as shown in Table 1.

### 3.2. The Composition of Chickpea Pasta

The chemical analysis showed that raw chickpea pasta was characterized by a moisture content of 9.5%. It contained 53.5 g of carbohydrates, 1.9 g of fat, 21.0 g of protein, 11.5 g of dietary fiber, and 2.6 g of ash per 100 g. It was observed that the content of resistant starch changed with the processing methods. The highest RS content was found in cooled and reheated pasta (3.65 g/100 g). This was higher than the RS amount in freshly cooked pasta and chickpea pasta that had been cooled for 24 h (1.83 g and 3.43 g/100 g, respectively).

### 3.3. Glucose Response to Tested Chickpea Pasta

**Figure 1:**
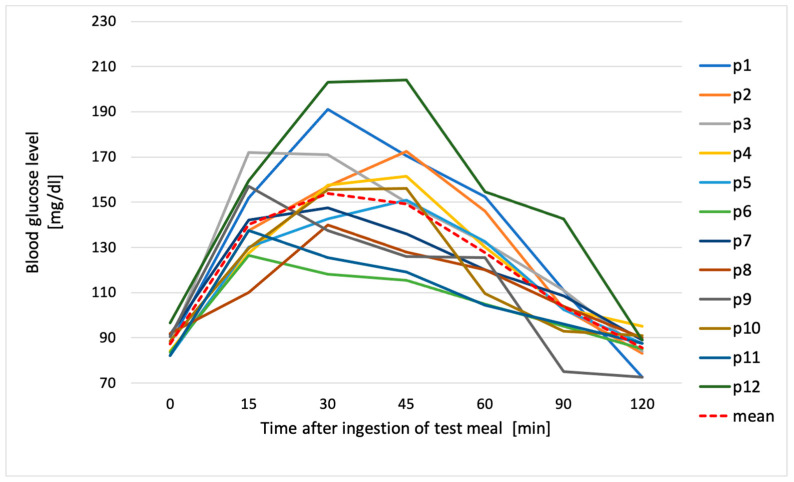
Individual glycemic responses and the average glucose value after ingestion of reference food (50 g of glucose); p1–p12—participant numbers.

All participants had normal fasting blood glucose, ranging from 70 mg/dL to 99 mg/dL. Blood glucose 120 min after the ingesting of glucose solution was also optimal (<140 mg/dL) in all subjects when averaged (85.5 ± 6.9 mg/dL). The highest glycemic peak after ingestion of the glucose solution varied among the subjects, as shown in Figure 1. In the largest number of subjects (41.7%), this peak occurred at 45 min after the ingestion of the solution; in 33.3% of subjects, it occurred at 15 min; and in the remaining 25%, it occurred after 30 min.

**Figure 2:**
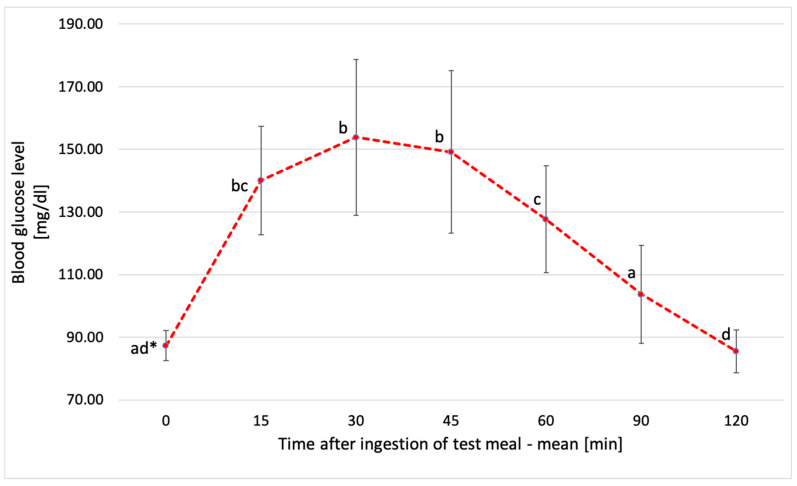
Mean blood glucose levels of all participants after consumption of glucose solution. * Values with different lowercase letters are significantly different. ANOVA, Tukey’s Test, *p* < 0.05. Error bars are SD.

The average blood glucose level of all participants after consuming the test meal reached its highest peak of 154 ± 24.9 mg/dL at 30 min. In the following 15–30 min intervals, a significant decrease in glucose levels was observed. The lowest value of 85.5 ± 6.9 mg/dL was found at the 120th minute of measurement, as shown in Figure 2.

**Figure 3:**
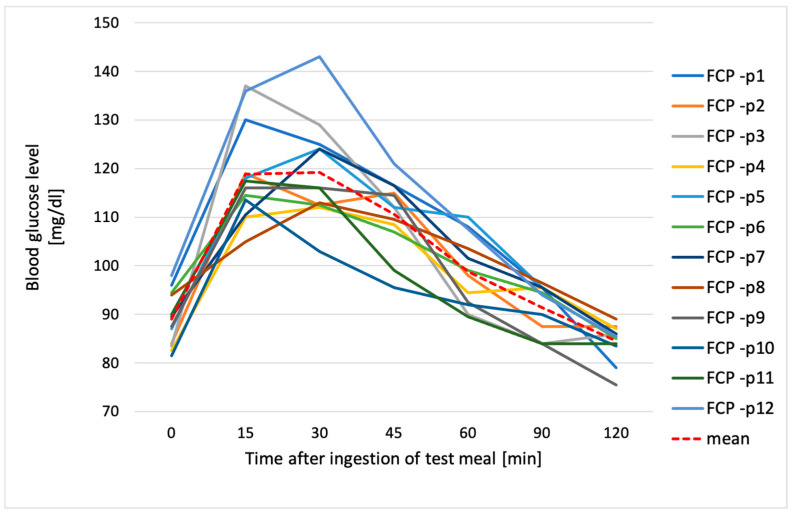
Blood glucose levels of all participants after consumption of fresh chickpea pasta (FCP); p1–p12—participant numbers.

**Figure 4:**
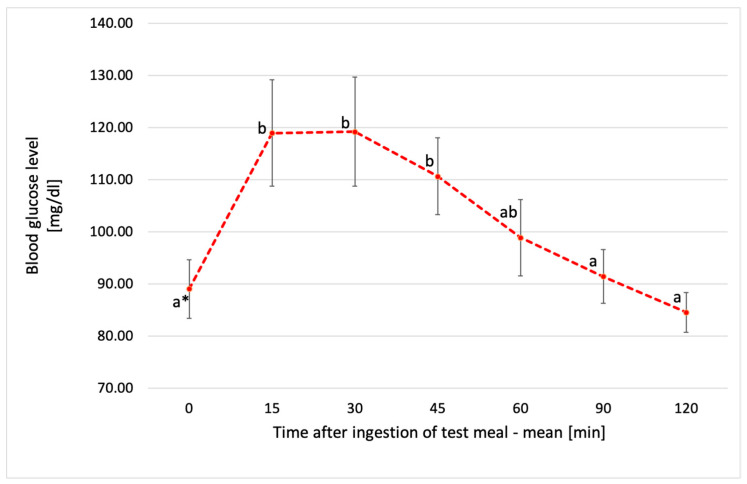
Blood glucose levels after consumption of fresh chickpea pasta (FCP)—mean value. * Values with different lowercase letters are significantly different. Absolute differences between sum of ranks, *p* < 0.05. Error bars are SD.

**Figure 5:**
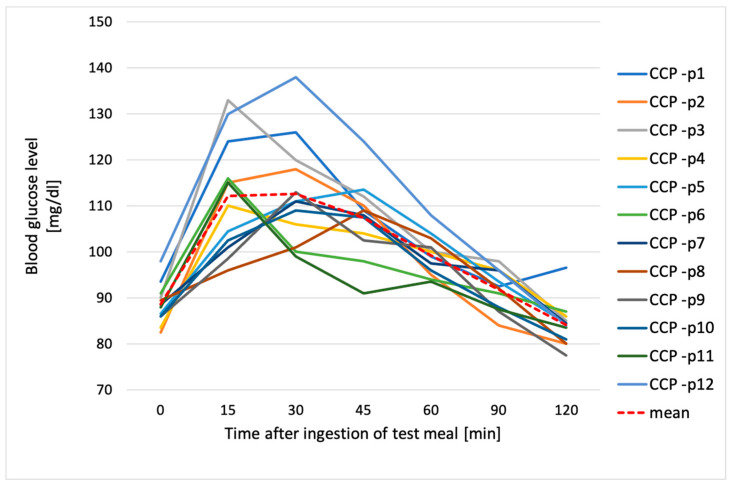
Blood glucose levels after consumption of cooled chickpea pasta (CCP); p1–p12—participant numbers.

**Figure 6:**
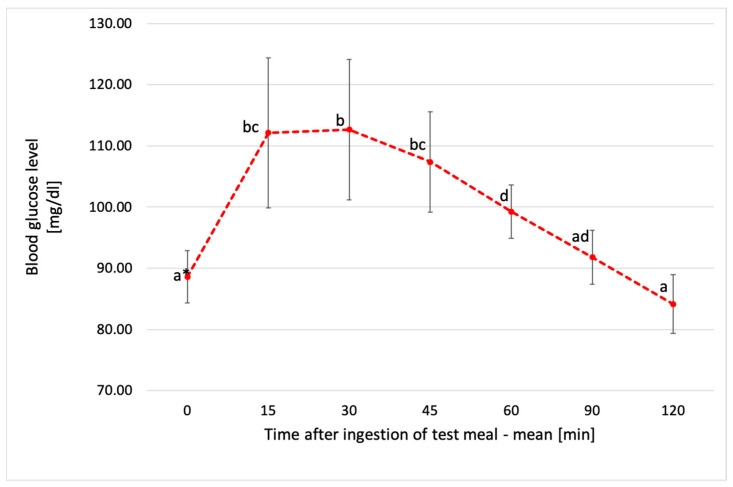
Blood glucose levels after consumption of cooled chickpea pasta (CCP)—mean value. * Values with different lowercase letters are significantly different. ANOVA, Tukey’s Test, *p* < 0.05. Error bars are SD.

Figure 3, Figure 4, Figure 5 and Figure 6 show individual and average blood glucose values after consuming a defined serving of tested chickpea pastas. For both FCP and CCP, the shape of the glycemic curve and the glycemic peak after intake of pasta varied among the subjects. In regard to FCP, it was observed that for the majority of participants (58.3%), this peak occurred 15 min after consumption, while for the remaining 41.7% of individuals, it was reached after 30 min after pasta intake (Figure 3). After consuming FCP, mean blood glucose levels rose steadily, peaking at 119 ± 10.5 mg/dL at 30 min (Figure 4).

**Figure 7:**
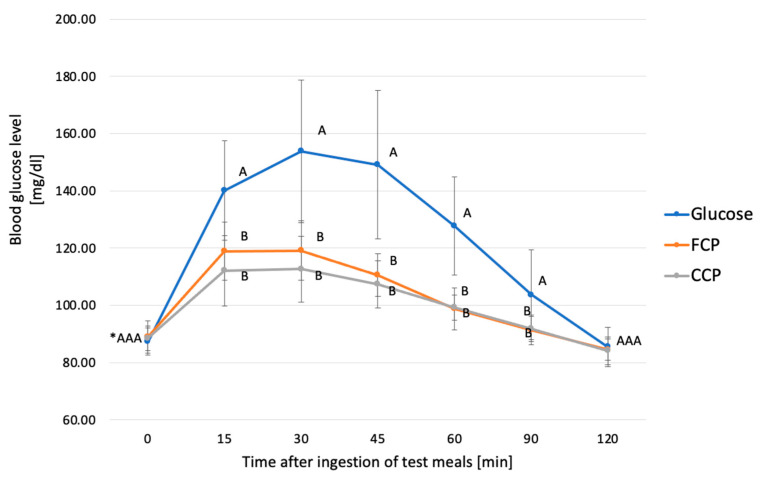
Postprandial glucose response to glucose solution (Glucose), fresh chickpea pasta (FCP), and cooled chickpea pasta (CCP). * Values with different uppercase letters at time points are significantly different. ANOVA, Tukey’s Test, *p* < 0.05. Error bars are SD.

The glycemic response after ingestion of CCP varied among individuals. However, it was shown that 41.7% of participants reported the highest blood glucose levels 30 min after eating the test meal, 33.3% of subjects after 15 min, and the remaining 25% of subjects 45 min after pasta intake (Figure 5). In the case of CCP, the highest average glucose concentration of 113 ± 11.5 mg/dL was shown at 30 min after ingestion, with a further decrease to a final value of 84 ± 4.8 mg/dL at 120 min (Figure 6). Moreover, the highest glycemic peak in both pastas tested (FFC and CCP), as well as after ingestion of the glucose solution, occurred 30 min after consumption. However, in the case of the glucose solution, it was significantly higher from both fresh and chilled pasta (*p* < 0.05), as shown prominently in Figure 7.

**Figure 8:**
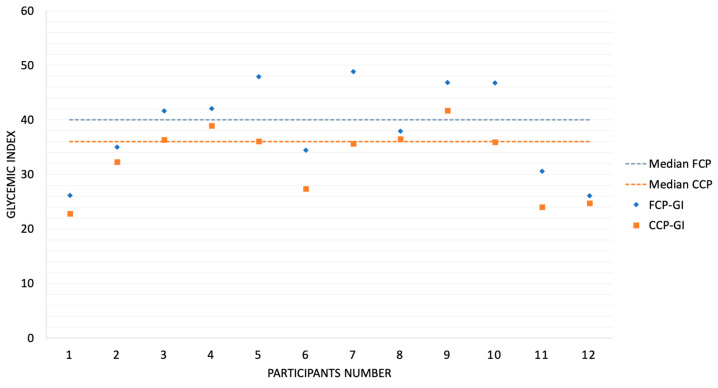
Comparison of the glycemic index (GI) and median value of fresh (FCP) and cooled chickpea pasta (CCP).

The area under the glycemic curve was significantly different between the FCP (1556.1 ± 456.9 mg/dL/min) and the CCP (1327.9 ± 414.8 mg/dL/min) pasta compared to the standard (4145.4 ± 1412.4 mg/dL/min) (*p* < 0.05). The GI values of both chickpea pastas were below 55, which corresponds to a low glycemic index. There was a significant difference (*p* = 0.0022) between the GI of FCP (GI = 39) and CCP (GI = 33). The calculation of the glycemic index (GI) as an average takes into account individual glycemic responses, but the final result represents an overall mean, which facilitates the comparison of different products. Individual GI results for all participants and the median are shown in Figure 8.

### 3.4. Sensory Profile of Tested Food

A sensory analysis revealed the consumer acceptability of both FCP and CCP chickpeas pasta. There were no significant differences between the FCP and the CCP pasta in terms of visual appearance, taste, smell, texture, and overall acceptability (Table 2). According to the respondents, both pastas had a similar taste.

## 4. Discussion

The current study was designed to evaluate the effect of cooling previously cooked chickpea pasta on resistant starch content, the glycemic index, and the postprandial glycemic response in healthy individuals. To the best of our knowledge, this study is the first to demonstrate that cooling and reheating 100% chickpea pasta had a positive effect on the postprandial glucose response. Our results support the common conception of a reduced postprandial glycemic response induced by cooling and reheating pasta and confirm that such a strategy may also be effective for 100% vegetable pasta. Similar research questions have been analyzed by other researchers. However, they have used pasta derived from different legumes (faba, green and yellow pea, lentil, black-gram) [25,26], mixed legume/wheat pasta [27,28], or conventional pasta [29] to examine the postprandial blood glucose response to different pasta compositions and different cooking methods. The current study analyzed the postprandial glucose level of 100% chickpea pasta, which is widely available on the market.

Pasta (mainly made from cereals) is a staple, very popular, and affordable food for both children and adults. It is easy to prepare, safe to store, and has a long storage life. Pasta made from 100% chickpea flour is characterized by a lower digestible starch content and higher vegetable protein and dietary fiber content than conventional cereal pasta. According to Saget et al. [30], cooked chickpea pasta is 2.6 times more nutrient-dense than durum wheat. It contains 1.5 times more protein, 3.2 times more dietary fiber, and 8 times more essential fatty acids than cooked durum wheat pasta per kcal energy content. Chickpea protein is rich in lysine, which is a limiting amino acid in cereals, and dietary peptides from the protein of chickpeas are gaining more attention [31]. Studies have shown that diets containing chickpea meals result in improvements in the serum lipid profile (total and low-density lipoprotein cholesterol levels), insulin sensitivity, and lipid peroxidation [32,33,34]. Furthermore, chickpeas are low in the nutrients that are commonly consumed in excess, such as saturated fat, added sugar, and sodium [35]. Replacing cereals with legumes in staple foods such as pasta can be a simple strategy through which to improve, simultaneously, the environmental sustainability and nutritional quality of food chains [30]. Legume-based pasta, including chickpea pasta, has also been studied as an alternative to gluten-free pasta to increase the nutritional profile of pasta through high fiber and protein content [26,36].

The processing of starchy products, i.e., changes in temperature, pressure, or water content, results in structural changes in starch molecules. The process of cooking induces significant changes in the structure and properties of starch, which plays a crucial role in determining the digestibility of starchy foods and the glycemic response. During cooking, starch granules absorb water and undergo gelatinization, a process in which the crystalline structure of starch is disrupted as it swells and leaches amylose and amylopectin into the surrounding water. This disruption of the crystalline structure of starch increases its digestibility by enhancing the accessibility of digestive enzymes to the starch molecules [37,38]. One of the key factors affecting the degree of starch gelatinization is cooking temperature and time. As the temperature increases, the gelatinization process accelerates, leading to a more complete breakdown of the starch granules. This can increase the glycemic response and GI of foods, as more easily digestible starch is available for the rapid release of glucose into the bloodstream after ingestion. In addition, the degree of gelatinization is also affected by the moisture content and specific type of starch present in the food matrix, the ratio of amylose to amylopectin, and other factors [39]. According to the applied factors, the amylose/amylopectin ratio, as well as the length of the amylose chains, linearization, or the retrogradation process, may change [40]. The formation of resistant starch (RS) during the cooling of chickpea starch involves the complex interplay of gelatinization, retrogradation, and the inherent properties of the starch’s amylose and amylopectin components. Chickpea starch, characterized by a high amylopectin-to-amylose ratio, undergoes gelatinization upon heating, where the starch granules swell and the crystalline structure disintegrates, allowing amylose and amylopectin to dissolve into the surrounding aqueous medium. As the starch gel cools, retrogradation occurs, characterized by the reassociation and recrystallization of these polysaccharides. Amylose, due to its linear structure, forms more-ordered crystalline regions, while amylopectin contributes to a less-ordered crystalline network. The extensive retrogradation in chickpea starch results in the formation of RS, specifically retrograded starch (RS3), which is less susceptible to enzymatic digestion due to its crystalline structure [41,42].

In our study, the RS content of chickpea pasta increased significantly as a result of cooling for 24 h at 4 °C. In a study by Johnson et al., similar findings were shown because the cooling process of lentils induced the synthesis of RS from gelatinized starch, and RS increased after cooling [43]. The effect of cold storage on the RS content of starchy products was also studied by Sonia et al. In their study, cold storage of rice for 10 h, as well as for 24 h, and reheating resulted in an increase in RS content compared to freshly cooked rice [13]. Sonia et al. also determined the area under the glycemic curve, which was significantly lower after cooling and reheating than for the freshly cooked product [13]. The conclusions of our study are similar because the area under the curve (AUC) of fresh pasta was 1556.1 ± 456.9 mg/dL/min, while for cooled pasta, it was 1327.9 ± 414.8 mg/dL/min. Another study by Nakamura et al. compared blood glucose levels after consuming plain rice and rice with increased RS content and also confirmed significantly lower postprandial glucose levels after consuming rice with higher RS content [44]. However, not all studies indicate that there is a significant effect of cooling starch products on lowering postprandial glycemia [45,46]. Our study showed a beneficial effect of using a specific thermal modification of chickpea pasta—namely, cold storage after pre-cooking—on postprandial glucose levels in healthy subjects.

The cooling process of starchy products leads to the formation of type-3 resistant starch, thus reducing the digestible carbohydrate content of the food [40]. The cooling process affects the physical properties of the starch granules. As the temperature drops, the reformation of crystalline regions within the starch granules leads to a more complex and less accessible starch matrix. This structural change slows the rate of starch digestion and absorption, contributing to a reduction in the glycemic index (GI) of the legumes [47]. Due to this effect, it is possible to obtain a lower GI in starchy products. Our study showed a significantly (*p* < 0.05) lower GI of cooled pasta (GI = 33) compared to fresh pasta (GI = 39). Other researchers compared the GI of processed and cooled chickpeas and showed that the GI of chickpeas consumed after cooking and immediate consumption was 34.0 ± 4.0, and after cooking and storage (at room temperature), it was 22.0 ± 2.0, and it was also 22.0 ± 2.0 after cooking, storage (20 °C), and heating in a microwave oven. The same authors found that cooked beans, stored in the fridge and reheated, also had a lower GI than beans that were consumed immediately after cooking [48]. Legume starches have been known to exhibit a lower GI than cereal or tuber starches due to high levels of amylose and strong interactions between amylose chains [49]. In one study by Yadav et al. (2009), the researchers used the technique of repeatedly heating and cooling starchy products, which also showed an increase in RS content; however, three cooling and heating cycles were used, and each cycle lasted 24 h, which is not possible for practical use considering the safety of the product for consumption [47]. For comparison, the current study was conducted with human subjects; therefore, the priority was to preserve the microbiological safety of the meals tested, as well as the possibility of future applications of the procedure in practice. Therefore, the test meals were cooled for 24 h, which is a common technique in households.

The sensory analysis of chickpea pasta in our study revealed that the cooling–reheating process did not affect the sensory attributes of chickpea pasta. Participants similarly perceived both fresh pasta (FCP) and cooled and reheated pasta (CCP). In general, the overall acceptability of CCP pasta was similar to that of FCP pasta. The results showed no significant differences in parameters such as appearance, taste, smell, or texture, and the overall acceptability of both freshly cooked and cooled and reheated pasta (*p* < 0.05). Similar results, related to the effect of cooling cooked white rice on postprandial glycemia [50], demonstrated no differences in sensory evaluation. Different but interesting results, i.e., better sensory evaluation, were obtained with cooled rice compared to freshly cooked rice in a study by Lu et al. [51]. Considering that the chickpea pasta was prepared without the inclusion of additives or flavor enhancers, and the sole variable was the cooling process, the outcomes appear promising for the application of comparable treatments in future studies.

Our study has several limitation. The study was conducted on a relatively small group of healthy individuals to assess their glycemic index (GI). However, this is in line with the international standards of GI examination. In order to formulate dietary recommendations for patients, it is essential to expand this scope of knowledge by including a larger number of participants. Our study primarily focused on assessing glycemia and the glycemic index. It would also be valuable to investigate insulin-related parameters, such as pre- and post-consumption insulin levels, as well as the HOMA-IR index, in future research. Additionally, it is important to evaluate the cooling process and its impact on the studied parameters in other products. Furthermore, similar studies should be conducted on a group of individuals with carbohydrate metabolism disorders to confirm whether the effect is the same as in healthy individuals.

## 5. Conclusions

The cooling process of chickpea pasta after it underwent cooking has a beneficial effect on postprandial glycemia in healthy people, and the cooled and reheated product had a lower glycemic index than the freshly cooked one. Cooling the chickpea pasta did not affect its organoleptic characteristics, such as taste, smell, appearance, or texture, and it did not have a worse overall rating than fresh pasta. Given the growing number of people suffering from carbohydrate metabolism disorders and the associated medical complications, it is reasonable to look for simple solutions to help prevent such conditions. As we have shown, refrigeration of cooked starchy foods such as chickpea pasta can be included in diet prevention and diet therapy for such disorders. This study highlights the potential of chickpea pasta to play an important role in the shift from animal protein to plant protein and higher dietary fiber consumption, which is critical to achieving more sustainable, healthy diets in industrialized countries. The affordability of chickpeas enhances their potential to play a key role in increasing the population’s intake of dietary fiber, which is a nutrient of public health concern. Future research should investigate the perception of chickpeas and barriers to intake among non-consumers.

## Figures and Tables

**Table 1:** Characteristics of the study subjects (mean ± standard deviation).

Characteristic	Total (n = 12)	Women (n = 8)	Men (n = 4)
Age (years)	25.3 ± 4.3	24.00 ± 4.0	28 ± 4.1
Weight (kg)	66 ± 10.0	61.38 ± 5.9	75.25 ± 12.1
Height (m)	1.7 ± 0.1	1.66 ± 0.1	1.81 ± 0.1
BMI (kg/m^2^)	22.5 ± 1.9	22.35 ± 2.2	22.91 ± 2.0
Fasting blood glucose (mg/dL)	87.33 ± 4.7	86.13 ± 3.5	89.75 ± 5.3

BMI—body mass index.

**Table 2:** Sensory profiles of tested chickpea pastas.

Attribute	FCP		CCP		*p* * FCP vs. CCP
	Mean ± SD	Minimum–Maximum	Mean ± SD	Minimum–Maximum	
Visual appearance	5.33 ± 0.9	3–6	5.58 ± 0.9	4–7	0.48
Flavor	4.5 ± 1.0	3–6	4.58 ± 1.4	1–6	0.36
Smell	4.25 ± 0.6	3–3	4.17 ± 0.6	3–5	0.72
Texture	4.08 ± 1.6	2–6	3.67 ± 1.4	1–6	0.21
Overall acceptability	4.83 ± 0.7	4–6	4.75 ± 1.2	2–6	1.00

FCP—fresh chickpea pasta; CCP—cooled chickpea pasta; * Wilcoxon signed-rank test.

## Data Availability

The original contributions presented in the study are included in the article. Further inquiries can be directed to the corresponding author.
